# Characteristics and Programme-Defined Treatment Outcomes among Childhood Tuberculosis (TB) Patients under the National TB Programme in Delhi

**DOI:** 10.1371/journal.pone.0013338

**Published:** 2010-10-12

**Authors:** Srinath Satyanarayana, Roopa Shivashankar, Ram Pal Vashist, Lakhbir Singh Chauhan, Sarabjit Singh Chadha, Puneet Kumar Dewan, Fraser Wares, Suvanand Sahu, Varinder Singh, Nevin Charles Wilson, Anthony David Harries

**Affiliations:** 1 Centre for Operational Research, International Union against Tuberculosis and Lung Diseases (The Union), Paris, France; 2 International Union against Tuberculosis and Lung Diseases (The Union), South East Asia Regional Office, New Delhi, India; 3 Centre for Community Medicine, All India Institute of Medical Sciences, New Delhi, India; 4 State Tuberculosis Office, Government of Delhi, Delhi, India; 5 Central Tuberculosis Division, Directorate General of Health Services, Ministry of Health and Family Welfare, Government of India, New Delhi, India; 6 Office of the World Health Organisation Representative to India, World Health Organisation, New Delhi, India; 7 Department of Paediatrics, Lady Hardinge Medical College, New Delhi, India; University of Witwatersrand, South Africa

## Abstract

**Background:**

Childhood tuberculosis (TB) patients under India's Revised National TB Control Programme (RNTCP) are managed using diagnostic algorithms and directly observed treatment with intermittent thrice-weekly short-course treatment regimens for 6–8 months. The assignment into pre-treatment weight bands leads to drug doses (milligram per kilogram) that are lower than current World Health Organization (WHO) guidelines for some patients.

**Objectives:**

The main aim of our study was to describe the baseline characteristics and treatment outcomes reported under RNTCP for registered childhood (age <15 years) TB patients in Delhi. Additionally, we compared the reported programmatic treatment completion rates between children treated as per WHO recommended anti-TB drug doses with those children treated with anti-TB drug doses below that recommended in WHO guidelines.

**Methods:**

For this cross-sectional retrospective study, we reviewed programme records of all 1089 TB patients aged <15 years registered for TB treatment from January to June, 2008 in 6 randomly selected districts of Delhi. WHO disease classification and treatment outcome definitions are used by RNTCP, and these were extracted as reported in programme records.

**Results and Conclusions:**

Among 1074 patients with records available, 651 (61%) were females, 122 (11%) were <5 years of age, 1000 (93%) were new cases, and 680 (63%) had extra-pulmonary TB (EP-TB)—most commonly peripheral lymph node disease [310 (46%)]. Among 394 pulmonary TB (PTB) cases, 165 (42%) were sputum smear-positive. The overall reported treatment completion rate was 95%. Similar reported treatment completion rates were found in all subgroups assessed, including those patients whose drug dosages were lower than that currently recommended by WHO. Further studies are needed to assess the reasons for the low proportion of under-5 years of age TB case notifications, address challenges in reaching all childhood TB patients by RNTCP, the accuracy of diagnosis, and the clinical validity of reported programme defined treatment completion.

## Introduction

Childhood tuberculosis (TB) has traditionally had a lower priority than adult TB within National TB Programmes (NTPs), because it is largely non-infectious, cases have been thought to be few, and the assumption that effective control of adult TB could prevent childhood TB. In many countries with high TB incidence, however, childhood TB (i.e., TB among the population aged less than 15 years) constitutes a significant proportion (approximately 11–14%) of the TB case-load and under-5 mortality [1], [2]. Of the estimated 9.3 million annual incident TB cases in the world in the year 2007, at-least 1 million are estimated to be less than 15 years of age [3]. Children are susceptible to infection with *M. tuberculosis* in the community, at greater risk of progressing to active disease when infected at a very young age [4] and there are also well-documented cases of children acting as a source of infection within a community [5], [6], [7]. These considerations justify a focus on the proper management of childhood TB for the control of TB.

The World Health Organisation (WHO) and the Global Stop TB Partnership have strongly recommended that National TB Programmes (NTPs) take responsibility for the diagnosis and treatment of all TB patients, including children [8]. However, NTPs—especially in resource limited settings—face numerous challenges in ensuring accurate TB diagnosis along with access to a supervised, child friendly treatment. Although detection and isolation of *Mycobacterium tuberculosis* by culture remains the corner stone for diagnosis, quality assured diagnostic tests other than sputum smear microscopy may not be universally available under the NTPs. This makes the diagnosis of TB in children challenging as children most often have pauci-bacillary pulmonary TB resulting in smear-negative pulmonary TB; and also a higher proportion of childhood TB presents as extra-pulmonary TB (EP-TB). Standardised anti-TB treatment regimens using the combinations of the first line anti-TB drugs are recommended for the programmatic management of TB in children. However concerns have been expressed over the adequacy of drug dosages [4], [9], [10], [11], [12]. In addition to these concerns, NTPs also face operational challenges in ensuring that all diagnosed childhood TB patients are notified and treated under the programme, addressing issues related to drug logistic management, and achieving adherence to therapy for optimal treatment outcomes [13]. Amidst all these challenges, one of the first things a programme can do is to obtain and evaluate data already existing within the programme setting to identify priority areas for programmatic interventions. These include assessment of case notifications and treatment outcomes, stratified by age, gender, type and category of TB [14], [15].

India contributes approximately 21% to the global incidence of TB and shares the largest burden of this disease in the world. India's Revised National TB Control Programme (RNTCP), with a total TB case notification of more than 1.52 million cases, contributed approximately 24% to the global new and relapse TB case notifications in the year 2007. The program-defined treatment success rate among new TB patients registered under RNTCP in 2007 was more than 85% [3]. In 2008, at the National level, 79,779 patients (6% of the new cases) registered under RNTCP were aged less than 15 years [16]. There is, however, limited information on the basic demographic, clinical characteristics and programme defined treatment outcomes of these TB patients [17]. Furthermore, concerns have been raised on the adequacy of the RNTCP-recommended drug doses, which for some children on a milligrams per kilogram basis fall below that recommended by WHO in 2009 [18], [19], [20]. The primary objective of this exploratory operational research study was to describe the basic demographic, clinical characteristics and programme specified treatment outcomes of patients aged <15 years registered for TB treatment in Delhi. The study also assessed these treatment outcomes between patients treated with appropriate drug doses and those treated with drug doses below that recommended in WHO 2009 guidelines.

## Methods

### Study Setting

Delhi State in North India (population of 17.6 million people) has 24 RNTCP reporting districts with 36 TB units. Under RNTCP, one TB Unit is a basic programme management unit, caters to a population of approximately 500,000, and maintains a separate TB register for each unit. In 2008, a total of 49,905 patients were registered for treatment, of which 38,027 patients were new cases. Among new cases, 5461 (14.5%) were <15 years. Delhi was chosen for this study primarily because the state has the highest notification rate (32 cases per 100,000 population) of TB patients aged less than 15 years, in the country when compared to the National average (7 cases per 100,000 population).

### Diagnosis and treatment of TB in Children aged <15 years under RNTCP

**Figure 1:**
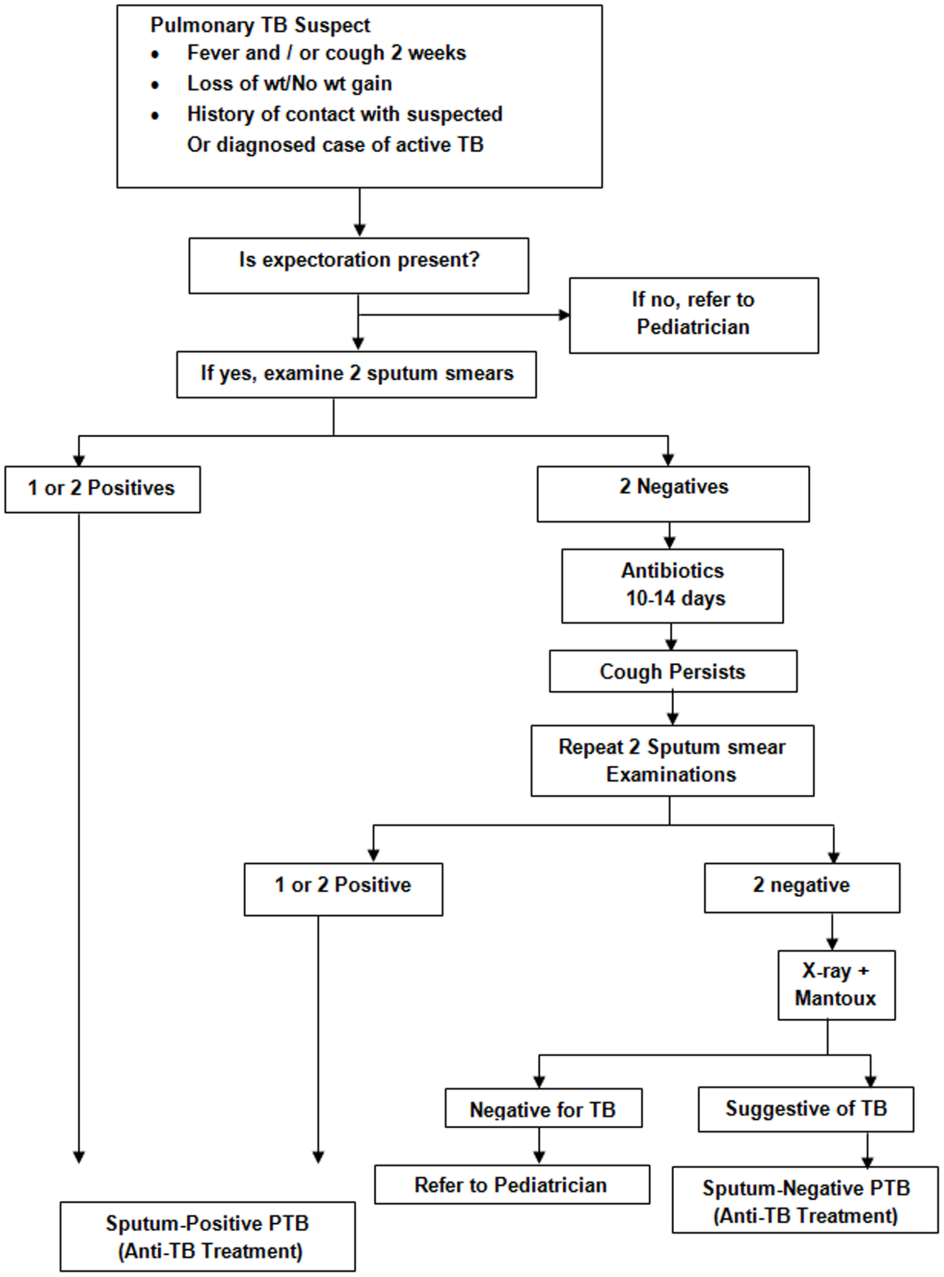
Diagnostic algorithm for paediatric pulmonary TB.

**Figure 2:**
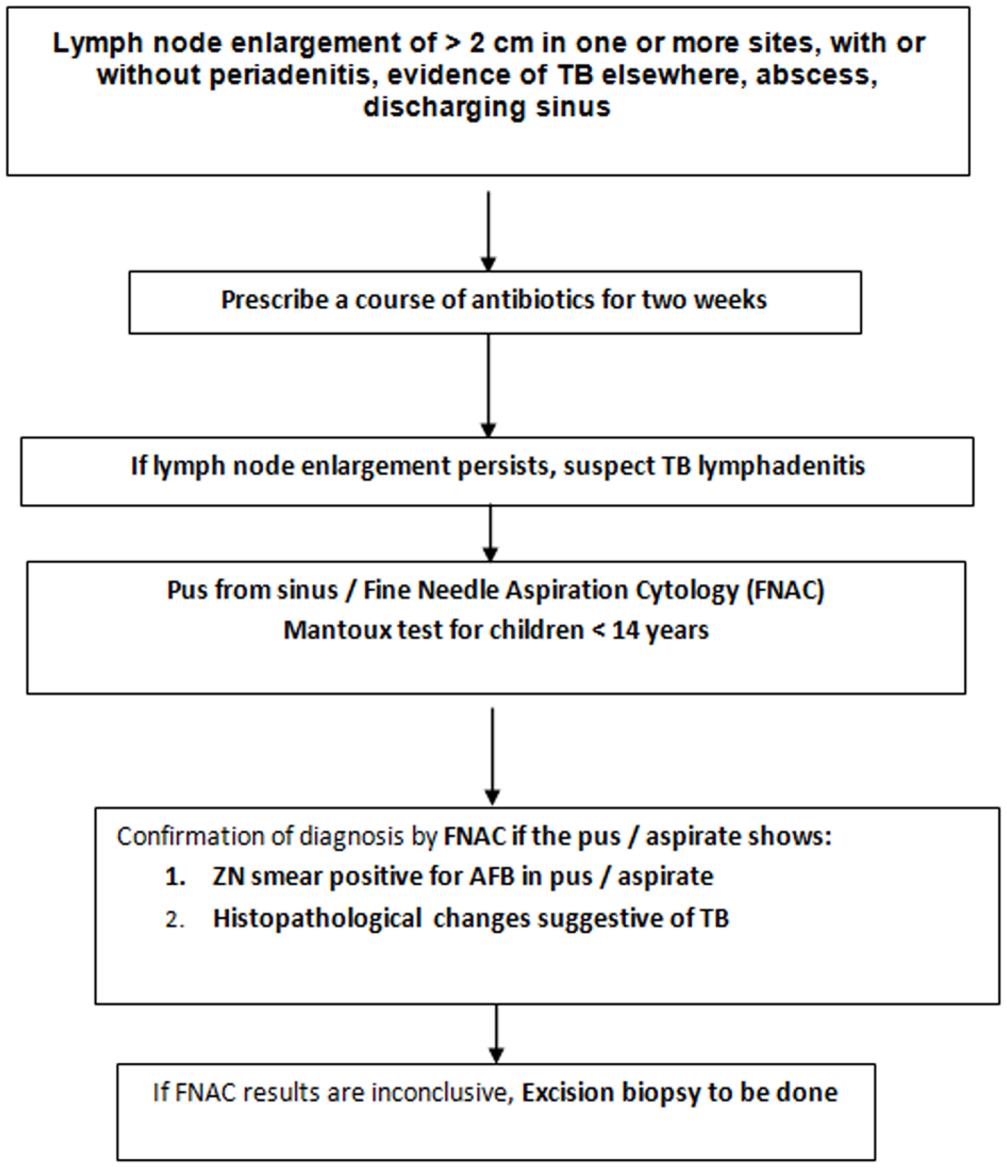
Diagnostic algorithm for peripheral lymph node TB.

**Figure 3:**
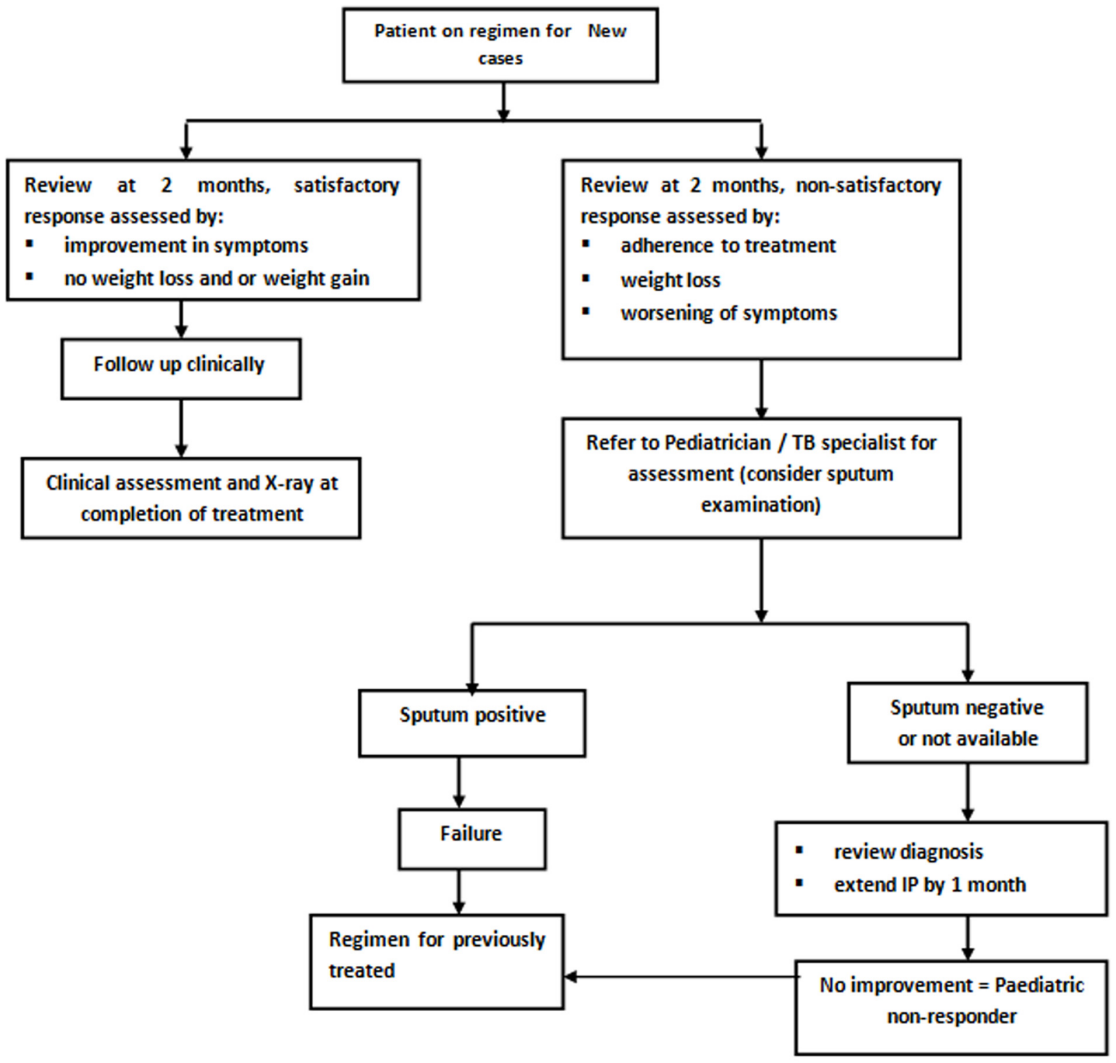
Algorithm for clinical monitoring of childhood TB patients.

Under RNTCP, Directly Observed Treatment Short-course (DOTS) is the recommended strategy for treatment of TB. The case definitions, disease classification, treatment regimens and treatment outcome definitions used by RNTCP are in line with standard WHO definitions [21], and are detailed in Table 1. In order to simplify the diagnosis and treatment of childhood TB, RNTCP in consultation with the Indian Academy of Paediatrics (IAP) has described criteria for suspecting TB among children, has separate algorithms for diagnosing pulmonary smear-positive TB (Figure 1) and peripheral TB lymphadenitis (Figure 2), and a strategy for treatment (Table 2) and monitoring patients who are on treatment (Figure 3) [22].

**Table 1:** Diagnosis and standard case definitions used under Revised National Tuberculosis Control Programme.

Disease classification
***Pulmonary TB, smear-positive*** *
•TB in a patient with at least two initial sputum smear examinations (direct smear microscopy) positive for Acid Fast Bacillus (AFB)
• Or: TB in a patient with one sputum smear examination positive for AFB and radiographic abnormalities consistent with active pulmonary TB, as determined by the treating medical officer
• Or: TB in a patient with one sputum smear specimen positive for AFB and culture-positive for Mycobacterium tuberculosis.
***Pulmonary TB, smear-negative***
• TB in a patient with symptoms suggestive of TB with at least three sputum smear examinations negative for AFB and radiographic abnormalities consistent with active pulmonary TB as determined by the treating Medical Officer followed by a decision to treat the patient with a full course of anti-tuberculosis treatment
• Or: diagnosis based on positive culture but negative AFB sputum smear examinations.
***Extra-pulmonary TB***
Extra-Pulmonary TB is TB of any organ other than the lungs, such as the pleura (TB pleurisy), hilar lymphadenopathy peripheral lymph nodes, intestines, genitourinary tract, skin, joints and bones, meninges of the brain, etc. Diagnosis should be based on culture-positive specimen from the extra-pulmonary site, histological, radiological, or strong clinical evidence consistent with active extra-pulmonary TB followed by decision of the treating MO to treat with a full course of anti TB therapy. A patient diagnosed with both pulmonary and extra-pulmonary TB should be classified as pulmonary TB (e.g., miliary TB)

Type of TB cases
***New*** **:** A TB patient who has never had treatment for TB or has taken anti-tuberculosis drugs for less than 1 month. A new case can be either sputum positive, sputum negative or extra-pulmonary.
***Relapse*** **:** A TB patient who had been declared cured or whose treatment had been completed by a physician, but who reports back to the health service and is now found to be sputum smear-positive.
***Treatment after default*** **:** A TB patient who received anti-tuberculosis treatment for at-least 1 month from any source and returns to treatment after having defaulted, i.e., not taken anti-tuberculosis drugs consecutively for at-least 2 months, and is found to be sputum smear-positive.
***Failure*** **:** Any TB patient who is smear-positive at 5 months after starting treatment. Failure also includes a patient who was treated with the Category III regimen but who becomes smear-positive during treatment.
***Transferred in*** **:** A TB patient who has been received for treatment into a TB Unit after starting treatment in another unit where s/he had been registered
***Others*** **:** A TB patient who do not fit into the above-mentioned types. Reasons for putting a patient in this type should be specified.

*The following modifications in the case definition have been made since 1st April, 2009.

1. The number of specimen required for diagnosis of smear-positive pulmonary TB is two, with one of them being a morning sputum specimen.

2. One specimen positive out of the two is enough to declare a patient as smear-positive TB.

**Table 2:** Treatment categories, Drug dosages and definitions of treatment outcomes.

1: Treatment categories and regiments			
Treatment category	Type of patients	Treatment regimens***	
		Intensive Phase	Continuation phase
Category 1	New sputum smear-positive PTB	2(H_3_R_3_Z_3_E_3_)	4(H_3_R_3_)
	New sputum smear-negative PTB, seriously ill*		
	New extra-PTB, seriously ill*		
Category 2	Sputum smear-positive relapse	2(H_3_R_3_Z_3_E_3_S_3_)+1(H_3_R_3_Z_3_E_3_)	5(H_3_R_3_E_3_)
	Sputum smear-positive treatment failure		
	Sputum smear-positive treatment after default		
Category 3	New sputum smear-negative, not seriously ill**	2(H_3_R_3_Z_3_)	4(H_3_R_3_)
	New extra-PTB, not seriously ill**		

2: Drug dosages						
Weight Bands	Isoniazid	Rifampicin	Pyrazinamide	Ethambutol	Streptomycin	Patient wise box used
<6kgs	10–15 mg/kg	10 mg/kg	30–35 mg/kg	30 mg/kg	15 mg/kg	
6–10Kgs	75 mg	75 mg	250 mg	200 mg	15 mg/Kg	1 PC†-13
11–17 Kgs	150 mg	150 mg	500 mg	400 mg	15 mg/Kg	1 PC†-14
18–25 Kgs	225 mg	225 mg	750 mg	600 mg	15 mg/Kg	1 PC-13+1 PC-14
26–30 Kgs	300 mg	300 mg	1000 mg	800 mg	15 mg/Kg	2 PC-14
>30 kgs	600 mg	450 mg	1500 mg	1200 mg	0.75 g	Adult boxes

3: Treatment outcomes
**Cured:** Initially sputum smear-positive patient who has completed treatment and had negative sputum smears, on two occasions, one of which was at the end of treatment.
**Treatment completed:** Sputum smear-positive patient who has completed treatment, with negative smears at the end of the intensive phase but none at the end of treatment. Or: Sputum smear-negative TB patient who has received a full course of treatment and has not become smear-positive during or at the end of treatment.
Or: Extra-pulmonary TB patient who has received a full course of treatment and has not become smear-positive during or at the end of treatment.
**Death:** Patient who died during the course of treatment regardless of cause
**Failure:** Any TB patient who is smear positive at 5 months or more after starting treatment. Failure also includes a patient who was treated with Category III regimen but who becomes smear positive during treatment.
**Defaulted:** A patient who has not taken anti-TB drugs for 2 months or more consecutively after starting treatment.
**Transferred out:** A patient who has been transferred to another Tuberculosis Unit/District and his/her treatment outcome is not known.

*In children, seriously ill sputum smear-negative PTB includes all forms of sputum smear-negative PTB other than primary complex. Seriously ill EP-TB includes TB meningitis (TBM), disseminated TB, TB pericarditis, TB peritonitis and intestinal TB, bilateral extensive pleurisy, spinal TB with or without neurological complications, genitourinary TB, and bone and joint TB.

**Not seriously ill sputum smear-negative PTB includes primary complex. Not seriously ill EP-TB includes lymph node TB and unilateral pleural effusion.

***Prefix indicates month and subscript indicates thrice weekly.

† PC = Product code. PC-13 is yellow coloured and PC-14 is orange coloured. Mg/kg = milligrams per kilogram body weight.

Sputum smears positive TB patient's under-go follow-up sputum examinations at the end of intensive phase and extended intensive phase (if IP extended), 2 months into continuation phase and at the end of treatment.

In brief, TB diagnosis is based on clinical features, smear examination of sputum where this is available, positive family history, tuberculin skin testing, chest radiography and histo-pathological examination as appropriate. The treatment strategy comprises three key components. First, as in adults, children with TB are classified, categorised, registered and treated with intermittent short-course chemotherapy (thrice-weekly therapy from treatment initiation to completion), given under direct observation of a treatment provider (DOT provider) and the disease status is monitored during the course of treatment (Figure 3). Second, based on their pre- treatment weight, children are assigned to one of 6 pre-treatment weight bands (<6kgs; 6–10 Kg; 11–17 Kg; 18–25Kg; 26–30Kg and >30Kgs). Patients with pre-treatment weight within the range of these individual weight bands are treated with a corresponding pre-fixed weight band dosage made available in individual patient-wise boxes. Patients weighing less than 6kg are treated with individualized dosages, and those weighing more than 30 Kg are treated using adult dosages. Third, uninterrupted good quality anti-TB drugs through “ready-to-use” patient wise boxes containing the patients' complete course of anti-TB drugs are made available to every registered TB patient according to programme guidelines [23].

TB is suspected in children seeking health care for their ailment by the treating physicians/paediatricians who may work in the public or non-public health centres. The RNTCP strongly recommends (although it is not mandatory) that these treating physicians/paediatricians both in the public and non-public sector should suspect, diagnose and treat childhood TB according to programme guidelines. There is a need for referral of diagnosed TB patients by the treating medical practitioners for the TB patients to get registered under the RNTCP and get into the RNTCP records and reports. If for any reason the TB cases are not referred to the programme then all such patients are not registered under the programme and are ‘missed’ in the notification system.

### Study population

All patients aged <15 years registered for TB treatment under RNTCP from 1^st^ Jan, 2008 to 30^th^ June 2008 in Delhi.

### Study design and sample size

We used a descriptive cross-sectional study design with retrospective review of existing programme records. Cluster sampling was used to select districts from which all eligible patients were included. The sample size was calculated for estimating programme defined “favourable” treatment outcomes (cured and treatment completed) with 95% confidence levels, with an absolute precision of ±3%. A sample size of 993 paediatric TB patients was calculated based on a) the overall proportion of successful treatment outcomes reported in Delhi during this period across all age groups (88%), b) the design effect of 2 for the cluster-sampling approach, and c) a 10% expected non-availability of information. RNTCP reporting districts (24 in number) were selected as the cluster unit. Expecting an average of 160 TB patients aged <15 years in each district from Jan–June, 2008, 6 clusters were randomly selected, with selection probabilities weighted by the Population Proportionate to the Size (PPS) sampling technique.

### Source of information, study variables and definitions

The sources of information were two RNTCP records namely, the TB register and treatment cards. The variables included: demographic (age and sex), clinical (disease classification, type of TB, site of EP-TB, pre-treatment weight) and treatment related (categorisation, treatment regimens, drug dosages, DOT provider type and location, treatment outcomes). We defined a “low milligram per kilogram body weight group” consisting of those patients whose treatment doses for isoniazid were below current WHO 2009 recommendations [19], [20], meaning those patients whose pre-treatment weights were at the upper end of the respective weight bands described above, i.e. 9–10 kg, 16–17 kg, 24–25 kg, or 29–30 kg. For the purpose of this study, all definitions are as per RNTCP definitions. Most of these cases would have also received lower than recommended dosages for other co-administered drugs as well.

Program defined outcomes were classified as ‘completed’ (which also includes cured) and ‘others’ (which includes deaths, default, failures, transferred out and not-recorded).

### Data entry and analysis

The data was extracted separately by two independent investigators. Data were entered into a pre-structured format created on Epi-info (version 3.2.2) and cross verified by two investigators and compared for consistency. All inconsistencies were resolved by referring to the records. The data were analysed in Epi-Info by complex sample analysis to account for cluster sampling methodology with districts as the primary sampling units. Variables are summarized by proportions and 95% confidence intervals (95% CI).

### Ethical Issues

As this study was a record review of the data collected under RNTCP, approval was obtained from the Central TB Division, Ministry of Health and Family Welfare, Govt. of India. The study protocol was also reviewed and approved by the Ethics Advisory Group of the International Union Against TB and Lung Disease. The activity was determined to be a retrospective programme evaluation of the implementation of national guidelines, hence individual patient consent was deemed unnecessary. Electronic databases created for this analysis were stripped of personal health identifiers and maintained securely.

## Results

### Demographic and clinical characteristics [Table 3]

**Table 3:** Basic Demographic and clinical characteristics of TB patients aged <15 years in Delhi (N = 1074).

Characteristics	n	%	(95% CI)
**Sex**			
Female	651	60.6	(54.7–66.3)
Male	420	39.1	(33.2–44.9)
Sex not recorded	3	0.3	(0.06–0.67)
**Age Groups**			
0- to <1-yrs-old	7	0.7	(0.13–1.18)
1 to <5 yrs-old	115	10.7	(5.9–15.4)
5 to 10 yrs-old	422	39.3	(35.8–42.7)
>10 to <15 yrs-old	530	49.3	(42.4–56.2)
**Pre-treatment RNTCP weight bands**			
≤6 kgs	6	0.6	(0.0–1.1)
>6–10 kgs	84	7.8	(4.2–11.5)
>10–17 kgs	198	18.4	(14.7–22.4)
>17–25 kgs	356	33.1	(29.3–37.6)
>25–30 kgs	139	12.9	(8.6–17.4)
>30kgs	280	26.1	(19.7–30.4)
not recorded	11	1.0	(0.0–2.1)
**TB Classification and type**			
Extra-Pulmonary	680	63.3	(60.5–66.1)
Pulmonary	394	36.7	(33.8–39.4)
**Type of TB**			
New Extra Pulmonary	641	59.7	(56.9–62.4)
New Smear-Negative Pulmonary	213	19.8	(15.5–24.1)
New Smear-Positive Pulmonary	146	13.6	(10.8–16.4)
Re-treatment Others	53	4.9	(4.0–5.8)
Re-treatment Relapse	11	1	(0.08–1.9)
Retreatment ‘Treatment After Default’	7	0.7	(0.2–1.0)
Re-treatment Failure	1	0.1	(0.0–0.3)
Not recorded	2	0.2	(0.0–0.6)
**Extra-Pulmonary TB site (n = 680)**			
Peripheral Lymph nodes	310	45.6	(37.8–53.4)
Abdominal	92	13.5	(6.8–20.1)
Pleural	77	11.3	(6.6–16.0)
Hilar Adenopathy	48	7.1	(2.3–11.7)
Bones/Joints	47	6.9	(3.7–10.0)
Brain/meninges	37	5.4	(3.0–7.8)
Others	36	5.3	(2.2–8.3)
Not recorded	33	4.8	(2.0–7.3)

Of 1089 TB patients aged <15 years registered in the 6 selected districts; treatment cards were available for 1074 patients (98.6%). There were more female than male children, and the majority of children were aged 5 years and above with just 11.4% being in the age group 0–5 years. There were only 7 children less than 1 year (less than 1% of all patients). Pre-treatment weight was documented in 99% of patients, and was less than 30 kg in 73% of the patients. Nearly two-thirds (63.3%) of patients were diagnosed with programme defined ‘EP- TB’, and peripheral lymph-node disease (45.6%) was the most common type, followed by abdominal (13.5%) and pleural TB (11.3%). Most patients (93.1%) were new (i.e., patients having received no anti-TB treatment or anti-TB treatment received for less than a month in the past), with the remainder being re-treatment TB cases (i.e., TB cases having received more than one month's anti-TB treatment in the past). Among the 394 programme defined ‘pulmonary TB cases’ (which also includes 11 miliary cases), 165 (42%) were sputum smear-positive (mean age 12 years, standard deviation 2.1 years). Among 72 re-treatment TB patients, 19 had sputum smear-positive pulmonary TB (classified by RNTCP definitions as relapse, failure and treatment after default); 13 patients had sputum smear-negative pulmonary TB and 40 had EPTB (These smear-negative pulmonary TB and EPTB are classified as per programme definitions as ‘Retreatment-others’).

### Treatment regimens and outcomes at the end of treatment [Table 4]

**Table 4:** Treatment characteristics and treatment outcomes of TB patients aged less than 15 years in Delhi (n = 1074).

Characteristics	n	%	95% CI
**Treatment category**			
Cat-1	748	69.6	(62.8–76.5)
Cat-2	76	7.1	(5.5–8.6)
Cat-3	245	22.8	(17.6–28.2)
ND-1*	1	0.1	(0.0–0.3)
ND-2**	1	0.1	(0.0–0.3)
Not recorded	3	0.3	(0.0–0.3)
**DOT centre type**			
Government health facility	755	70.3	(44.1–96.5)
Community Volunteer	167	15.5	(0.0–32.4)
Non Governmental Organisation	120	11.2	(0.7–21.6)
Private practitioner	16	1.5	(0.0–3.8)
Not recorded	16	1.5	(0.5–2.2)
**Treatment outcomes**			
Completed	899	83.7	(79.0–88.3)
Cured***	121	11.3	(7.4–15.0)
Defaulted	28	2.6	(0.6–4.6)
Death	12	1.1	(0.0–2.3)
Failure	6	0.6	(0.0–1.3)
Transferred Out	4	0.4	(0.0–0.7)
Not recorded	4	0.4	(0.0–1.0)

*ND-1 = Treated with Non DOTS regimen-1(2 months Streptomycin(S), isoinazid (H) and Ethambutol (E) and 10 months of H and E.

**ND-2 = Treated with Non DOTS regimen-2(12 months of H and E), ND-1 and ND-2 regimens are self administered non rifampicin containing regimen) used in exceptionally few cases.

***Only for pre-treatment smear-positive patients, if they completed the treatment and were smear-negative at the end of treatment and one other occasion during the course of treatment.

Almost all patients (99.5%) were categorised and prescribed RNTCP standardized short-course chemotherapy regimens. The majority of patients (92.4%) received treatment regimens for the new cases (category-1 or category-3), 7.1% of the patients received the re-treatment regimen (category-2), and the remaining 5 cases (0.5%) were prescribed either non-rifampicin containing self-administered regimens or the type of treatment was not recorded. The type of treatment centre from where the patients received observed treatment was recorded in 98.5% of the patients, with the majority of patients (755; 70.3%) receiving treatment from a government health facility, followed by a community volunteer or a non-governmental organisation facility. Standard programme defined treatment outcomes were recorded in in all but 4 patients; 95% of the patients were reported to have been successfully treated, i.e., completed or cured. Other outcomes [defaulted (n = 28), death (n = 12), failure (n = 6), transferred out (n = 4)] were noted in 4.3% of the patients. During the study, misclassification of treatment outcomes in the TB register was identified by the study investigators in 1.4% (n = 13), relative to the treatment cards and these were corrected during data entry. The median duration of treatment was 182 days (inter-quartile range 178–187 days) for patients, whose outcome was ‘treatment completion’ (78 doses)with the treatment regimen for new cases (category-1 and category-3), and 242 days (inter-quartile range 238–256 days) for patients who completed treatment (102 doses) with the regimen for retreatment cases (category 2).

### Association between Demographic and clinical variables to treatment outcomes

Program defined treatment outcomes were not significantly different among sub-groups of patients according to pre-treatment demographic and clinical variables (Table 5).

**Table 5:** Association between demographic and clinical characteristics with treatment completion for TB patients aged <15 years in Delhi.

Demographic and clinical characteristics	Programme specified treatment outcome*					
	Treatment completed			Other		
	n	%	95%CI	n	%	Total
**Sex (n = 1071)**						
Female	614	94%	(90.7–97.9)	37	6%	651
Male	403	96%	(93.4–98.4)	17	4%	420
**Age (in years)[n = 1074]**						
<5 years	154	96%	(90.6–99.5)	6	4%	160
≥5 to 10 years	367	96%	(93.8–98.1)	17	4%	384
>10 to <15 years	499	94%	(91.0–97.2)	31	6%	530
**RNTCP pre treatment weight bands [n = 1063]**						
≤10 Kgs	85	95%	(88.0–100)	5	5%	90
>10–17 Kgs	189	95%	(89.9–100)	9	5%	198
>17–25 kgs	339	95%	(92.8–97.6)	17	5%	356
>25–30 kgs	130	94%	(89.8–97.2)	9	6%	139
>30 kgs	270	96%	(93.3–99.5)	10	4%	280
**TB Classification [n = 1074]**						
Extra-Pulmonary	655	96%	(94.1–98.5)	25	4%	680
Pulmonary	365	93%	(88.8–96.4)	29	7%	394
**Type of TB [n = 1072]**						
New Extra Pulmonary	621	97%	(94.6–99.1)	20	3%	641
New Smear-Negative	201	94%	(88.7–100.0)	12	6%	213
New Smear-Positive	134	92%	(89.0–94.5)	12	8%	146
Re-treatment Others	48	91%	(82.3–98.8)	5	9%	53
Re-treatment Smear-Positive	16	84%	(60.1–100)	3	16%	19
**Extra-Pulmonary TB site [n = 647]**						
Peripheral Lymph node	302	97%	(94.8–100)	8	3%	310
Other sites	326	97%	(94.6–98.8)	11	3%	337
**DOT centre type [n = 1059]**						
Government health facility	718	95%	(91.8–98.3)	37	5%	755
Other DOT providers	291	96%	(92.6–98.8)	13	4%	304
**Treatment category [n = 1069]**						
Cat-1	713	95%	(92.9–97.7)	35	5%	748
Cat-2	68	89%	(80.8–98.0)	8	11%	76
Cat-3	237	97%	(92.9–100)	8	3%	245

*‘Treatment completed’ also includes sputum positive patients who were declared as cured and ‘others’ includes deaths, default, failures, transferred out and not-recorded.

There were 287 patients classified as “low milligrams per kilogram body weight group”, and 776 patients classified as “appropriate milligram per kilogram body weight group”. The proportion of patients with programme defined treatment completion rates was 95% (95% CI: 91.6% to 97.8%) in the low milligrams per kilogram weight group and 96% (95% CI: 94.1% to 98.4%) in the appropriate milligram per kilogram body weight group.

## Discussion

This study is one of the very few studies that reports data on the community-wide profile and treatment outcome of TB patients aged less than 15 years registered and treated under routine programme conditions in India. Such data are useful to understand the impact of the programmatic processes and assist in evidence-based programme policy formulation, planning and management. The programme defined treatment completion rate was >95%, which is reassuringly similar to treatment outcomes reported by other hospital-based studies [24], [25], [26], [27]. This suggests that the treatment strategy adopted by RNTCP in treating children with TB disease has been effective In this respect, the study findings however identifies certain priority areas that need to be addressed by the National and State health authorities.

First, patients aged less than 5 years constituted only 11% of the patients in this study despite the fact that rates of childhood TB are usually considered the highest among those aged 1–4 years [28]. Studies from the tertiary care setting in India have previously shown that patients aged less than 5 years constituted a much higher proportion (18–34%) [29] of total childhood TB cases. The demographic distribution of pediatric TB cases is unusual, and suggests that many TB patients aged less than 5 years are being missed by the programme due to non-diagnosis or treatment outside the programme setting. The reasons for these cases remaining outside the TB programme are speculative and must be ascertained in the future. The reasons may include poor access to diagnostic services (such as chest radiography, culture facilities, etc) or poor access to treatment services. The referral of paediatric cases, particularly children under 5 years of age, may not be as effective as it should be because these children may often have disseminated or severe disease. Paediatricians may often prefer to keep these children under their care for a more comprehensive management of their other attendant morbidities which cannot be managed under the programmatic conditions by the DOT provider. Further the programme does not provide patient wise boxes for very small children weighing less than 6 Kg. This further may be affecting the enrolment of children in the age group of 0–5 years.

Second, the majority of the patients treated had EP-TB and sputum smear-negative PTB. Only 15% of the patients were sputum smear-positive; this differs dramatically from adults, among whom approximately half of notified TB cases have sputum smear-positive PTB. This is similar to the findings from other studies [1], [4], [30]. RNTCP currently issues well-defined diagnostic algorithms for the diagnosis of sputum smear-positive PTB and TB lymphadenitis, which would have helped in the diagnosis of about 60% of the children in this study, but guidance for the diagnosis of pediatric TB for other forms of disease is limited. With respect to the diagnostic tools, currently, sputum smear microscopy [31] is the only quality assured diagnostic tool provided and available under the programme. Other methods for diagnosing childhood TB using—for example chest radiography or tuberculin skin-testing are not provided by the programme. The provision of radiography services are the responsibility of the respective State Government general health services and not the RNTCP. In relation to tuberculin skin testing, RNTCP has tried unsuccessfully to provide a supply of quality assured tuberculin. There are also no mechanisms existing to assess the quality of chest radiography or tuberculin test where available and when used. Under these circumstances, both under-diagnosis (especially in younger children) and over diagnosis remain a concern. Previous studies have shown that the diagnostic misclassification can be up to 15%–20% in children seeking health care in high TB-incidence communities with limited quality assured diagnostic tools [32], [33]. These findings suggest that the RNTCP should, in consultation with the relevant professional bodies, urgently make additional efforts to further develop algorithms and criteria for diagnosis and management of childhood TB within programmatic framework, particularly EP-TB beyond lymphadenitis. This will hopefully increase the confidence of the treating medical practitioners on the programme which may increase the number of TB cases (especially among very young children) notified to the programme.

Third, the programme defined treatment outcome ‘treatment completion’ is based on programmatic end points and not on the basis of well defined clinical criteria. The usage of programmatic terminology for reported treatment outcomes such as “completion” might be misleading when compared to clinical trial conditions. Whilst under clinical trial conditions, a successful response to treatment is validated against pre-specified criteria, what the programme terms as treatment completion is not subject to standardized validation. It is possible that some providers reported treatment as ‘completed’ on the basis of drug consumption, and may not have sufficiently assessed clinical response to treatment. The programme must consider outlining further clinical response criteria for characterizing the programme defined treatment outcome ‘treatment completion’.

Fourth, the programme defined treatment completion rates were similar among those treated with doses that were less than or in accordance the WHO recommendations. The anti-TB drug dosages and the regimens were formulated by RNTCP in consultation with the Indian Academy of Pediatricians, based on the treatment guidelines recommended by WHO in 2003 [9] [2]. However, WHO subsequently increased the dosages per kilogram recommended for children in its later revision of the treatment guidelines [19], [20], [34]. Concerns have been raised about under-dosing of children whose pre-treatment weight is at the higher end of the individual RNTCP pre-treatment weight bands (e.g, 9–10 kgs, 16–17 kgs, 24–25 kgs, 29–30 Kgs) [18] [12]. Our study shows that programme defined treatment completion rates were not different between those on the high end of the weight band with lower milligrams per kilogram dosing than currently recommended, as compared to those patients treated with WHO-recommended dosing. This finding, however, needs validation using more robust diagnostic and clinical outcome criteria and longer term follow-up, which is not routinely feasible in programme settings.

Lastly, In Delhi being a low HIV prevalence state (General Population prevalence of 0.25% [35]), the routine offer of counselling, voluntary testing of HIV status and recording of the status of HIV infection on the programme records for all TB patients was not the policy in 2008. Hence we could not ascertain the HIV status of this study cohort from the records. The programme has, however, revised its policy in 2009, including Delhi into the list of states in the country where counselling, voluntary testing and recording of HIV status on programme records for all TB patients has become a routine activity. The programme must use the opportunity provided by this revised policy to assess the relative contribution of HIV infection to paediatric notifications in Delhi.

### Limitations of the study

First, the study was retrospective in nature, and record keeping may have been sub-optimal. However, since the programme is supervised and monitored rigorously by programme managers and staff from multiple levels which includes periodic data validation and accuracy checks according to programme guidelines [36], we feel that major inaccuracies are unlikely.

Second, in this retrospective record review, we could not evaluate the validity of the diagnosis, or the adequacy of therapy at the end of treatment and long term treatment outcomes. Since the majority of patients had EP-TB and sputum smear-negative PTB, there are possibilities of misclassification of TB diagnosis and treatment outcomes. However, since Delhi is an urban centre with many tertiary care hospitals and as most of the childhood TB patients based on the programmatic observation are diagnosed by pediatricians and referred to the programme for treatment, the extent of misclassification of TB diagnosis, if any, will most likely be insignificant.

Furthermore, the sample size in our study may not have been large enough to have sufficient power to detect significant differences among different demographic and clinical subgroups. However, such small differences are not likely to be programmatically significant.

### Conclusions and recommendations

As measured by programme defined treatment outcomes, childhood TB patients in Delhi, across all groups notified under the RNTCP which follows WHO TB treatment guideline definitions, have high treatment completion rates. The demographic and clinical profile of registered childhood TB patients shows that they are mostly aged 5–15 years and with programme defined non-serious forms of TB. Further studies are needed to assess i) the reasons for the low proportion of under 5 years of age TB case notifications, identify and implement strategies to reach out to the cases missed by the programme and ii) the accuracy of diagnosis and treatment clinical response in various demographic and clinical subgroups, especially when programmatic definitions of ‘treatment completion’ are used.
